# Outcomes and Impact of HIV Prevention, ART and TB Programs in Swaziland – Early Evidence from Public Health Triangulation

**DOI:** 10.1371/journal.pone.0069437

**Published:** 2013-07-26

**Authors:** Cari van Schalkwyk, Sibongile Mndzebele, Thabo Hlophe, Jesus Maria Garcia Calleja, Eline L. Korenromp, Rand Stoneburner, Cyril Pervilhac

**Affiliations:** 1 The South African Department of Science and Technology / National Research Foundation Centre of Excellence in Epidemiological Modelling and Analysis (SACEMA), University of Stellenbosch, Cape Town, South Africa; 2 Strategic Information Department, Swaziland Ministry of Health, Mbabane, Swaziland; 3 Department of HIV/AIDS, World Health Organization, Geneva, Switzerland; 4 Department of Public Health, Erasmus MC, University Medical Center, Rotterdam, The Netherlands; 5 Independent consultant (rlstoneburner@gmail.com). Former Senior Advisor on Strategic Intelligence and Analysis, UNAIDS, Geneva, Switzerland; 6 Independent consultant (pervilhacc@gmail.com). Formerly at Department of HIV/AIDS. World Health Organization, Geneva, Switzerland; Alberta Provincial Laboratory for Public Health/ University of Alberta, Canada

## Abstract

**Introduction:**

Swaziland’s severe HIV epidemic inspired an early national response since the late 1980s, and regular reporting of program outcomes since the onset of a national antiretroviral treatment (ART) program in 2004. We assessed effectiveness outcomes and mortality trends in relation to ART, HIV testing and counseling (HTC), tuberculosis (TB) and prevention of mother to child transmission (PMTCT).

**Methods:**

Data triangulated include intervention coverage and outcomes according to program registries (2001-2010), hospital admissions and deaths disaggregated by age and sex (2001-2010) and population mortality estimates from the 1997 and 2007 censuses and the 2007 demographic and health survey.

**Results:**

By 2010, ART reached 70% of the estimated number of people living with HIV/AIDS with CD4<350/mm^3^, with progressively improving patient retention and survival. As of 2010, 88% of health facilities providing antenatal care offered comprehensive PMTCT services. The HTC program recorded a halving in the proportion of adults tested who were HIV-infected; similarly HIV infection rates among HIV-exposed babies halved from 2007 to 2010. Case fatality rates among hospital patients diagnosed with HIV/AIDS started to decrease from 2005–6 in adults and especially in children, contrasting with stable case fatality for other causes including TB. All-cause child in-patient case fatality rates started to decrease from 2005–6. TB case notifications as well as rates of HIV/TB co-infection among notified TB patients continued a steady increase through 2010, while coverage of HIV testing and CPT for co-infected patients increased to above 80%.

**Conclusion:**

Against a background of high, but stable HIV prevalence and decreasing HIV incidence, we documented early evidence of a mortality decline associated with the expanded national HIV response since 2004. Attribution of impact to specific interventions (versus natural epidemic dynamics) will require additional data from future household surveys, and improved routine (program, surveillance, and hospital) data at district level.

## Background

With increasing global momentum and HIV financing brought about with the “3 by 5” initiative [1,2] and creation of the Global Fund to fight AIDS, tuberculosis and malaria (GF) in 2002, low- and middle-income countries have rapidly scaled-up HIV Testing and Counseling (HTC) as entry point to treatment and care, as well as Antiretroviral Therapy (ART), HIV-TB and Prevention of Mother to Child Transmission (PMTCT) programs. More recent initiatives by the World Health Organization (WHO), the Joint United Nations Programme on HIV/AIDS (UNAIDS) and partners have set ambitious goals and targets to achieve universal access to comprehensive HIV care by 2015 [3–5].

To monitor and evaluate the health impact of intensified program efforts, WHO, UNAIDS, the GF and partners provide Monitoring and Evaluation (M & E) guidance for national HIV/AIDS programs to report on policy development, implementation and programmatic progress of HTC, ART, HIV-TB and PMTCT services [3–9]. These Global Health Initiatives developed guidance on ‘triangulating’ a combination of relevant existing data sources to understand national and local HIV epidemics and evaluate program outcomes and impact [10,11]. The triangulation approach was initially developed and tested in two countries with high HIV prevalence and relatively advanced program responses, Botswana [12–15] and Malawi [16–19]. Other examples of successful HIV data triangulation include Ukraine, Republic of Moldova and Estonia [20,21].

This article presents the results of a data triangulation for key HIV and TB interventions in Swaziland [11,22]. A program progress evaluation using triangulation is timely for Swaziland for three reasons. First, Swaziland is among the worst-hit countries in the world, with an estimated national HIV prevalence of 26% among adults 15 to 49 years in 2010. Second, the country started its HIV response early, as illustrated by the inclusion of HIV/AIDS in the National Development Plan in the early 1990s [23]. Third, Swaziland has regularly reported program outcomes since the onset of its priority national treatment program in 2004 [24]; yet its routine program and surveillance data continue to have limitations. This study thus aimed to assess outcome and impact, using existing data on key programmatic indicators, and to build evaluation capacity through a country-owned process.

### Swaziland demography

The Kingdom of Swaziland is a Southern African country with an estimated population of 1.2 million people in 2010. About 77% of the population lives in rural areas [25]. Women of childbearing age (15-49 years) make up 26.2% of the population [26] and about 40% of the population are children under 15 years of age. The total fertility rate is estimated at an average of four births in a woman’s lifetime as of 2007, compared with 6.4 births in 1986. The life expectancy at birth has drastically worsened over the two decades of HIV spread, from 56 years in 1986 to 45.1 years in 2007. Antenatal care (ANC) attendance in Swaziland is high: 97% of women make at least one ANC visit during their pregnancy and 79% make at least four ANC visits [26].

### HIV and Tuberculosis Programs

A national response to the HIV epidemic was documented as early as 1987. The National Emergency Response Council on HIV and AIDS (NERCHA), established in 2003, produced two National multi-sectoral HIV and AIDS Strategic Plans (NSP I 2003-2005 and NSP II 2006-2008) that aimed to address HIV prevention through e.g. behavioral change communication and promotion of condom use. A 2008 review of the NSP II resulted in the development of the National multi-sectoral Framework for HIV and AIDS (2009-2014) that proposes an evidence-based management approach [27].

The Antiretroviral Therapy (ART) program was launched in 2004 by the Ministry of Health and Social Welfare. Scale up was implemented in three phases: the first phase established ART services in Swaziland’s six major hospitals; the second phase expanded ART delivery to five health centers and some private clinics; in the third phase more private as well as public clinics were added. The number of facilities providing HIV care increased from 19 in 2006 to 114 in 2010 after ART care was decentralized to peripheral clinics serviced by staff from the main clinics in 2008. In parallel with this increased service availability, new treatment guidelines in 2009 expanded the criteria for patient eligibility for enrolment onto ART to all HIV/TB co-infected patients, all HIV-infected children younger than 24 months and all people living with HIV/AIDS (PLWH) with a CD4 count below 350/mm^3^, following an earlier 200/mm^3^ threshold.

A Voluntary Counseling and Testing (VCT) program has been implemented since 2002. Since 2007, HIV Testing and Counseling (HTC) was implemented with priority, including a shift from patient-initiated to provider-initiated counseling and testing, as part of the standard of care in health facilities offered to all patients and clients.

Swaziland instituted PMTCT services within its maternal, new-born and child health services in 2003. Coverage of PMTCT services was progressively expanded, from 3 clinics initially to 150 Health facilities (88% of facilities providing ANC) by 2010 [28]. High coverage of PMTCT services among HIV-infected pregnant women is facilitated by the high antenatal care attendance. Since 2003 focus was on the reduction of mother-to-child transmission among HIV-infected women through single-dose Nevirapine, but in recent years more attention is given to primary prevention of HIV, prevention of unintended pregnancies among HIV-infected women and provision of treatment, care and support for HIV-infected women and their families [29,30].

To determine the HIV status of exposed infants, the country started deoxyribonucleic acid polymerase chain reaction (DNA PCR) testing using dried blood spot (DBS) in 2007, allowing for early infant diagnosis of HIV from as early as 6 weeks of age. As of 2010, 131 (49%) of all facilities are capable of collecting DBS samples for HIV testing using DNA PCR. All DBS samples are sent to the National Reference Laboratory for DNA PCR tests, which performs about 860 tests per month [28].

Tuberculosis control has historically been the responsibility of the national TB center. In 2006, national coordination and M & E of TB control was transferred to the newly established National TB Control Program (NTCP), while the national TB center kept responsibility for clinical duties. Collaboration between the NTCP and the Swaziland National AIDS Program (SNAP) through the National TB/HIV Steering Committee was established in 2007, facilitating sharing of information and consolidating efforts in the management of HIV/TB patients. As of 2010, 51 TB treatment initiation sites provide both TB treatment and ART for TB patients co-infected with HIV. In response to the emerging threat of multidrug-resistant and extensively drug-resistant tuberculosis (MDR/XDR-TB), a national referral hospital for MDR-TB became operational in 2009 and four more regional hospitals initiate treatment and manage MDR-TB since 2011.

All primary healthcare services in Swaziland are free to patients, including antiretroviral treatment, tuberculosis treatment and antenatal care. Patients however need to pay for all hospital admissions, irrespective of cause.

## Methods

### HIV data triangulation in Swaziland

The current study aimed to address the key questions listed in Table 1, which were agreed between the Ministry of Health Monitoring and Evaluation Team and key partners in a consensus workshop (2011), as representing the priority questions for Swaziland’s national HIV/AIDS programs.

**Table 1:** Key questions, by intervention.

**1. Common across all interventions**
How has the coverage of key interventions evolved from 2003 to 2010?
Are available data of such completeness, representativeness and quality (including consistency or changes over time) as to allow assessment of impact of interventions and trends in the national population?
**2. HIV Testing and Counseling (HTC)**
Is the uptake of HTC associated with enrolment on and coverage of ART over time?
**3. ART**
Given coverage of ART (in numbers and as proportion of HIV-infected in need), is it plausible that ART has reduced adult and/or infant case fatality and mortality among patients admitted in hospitals?
Given coverage of ART (in numbers and as proportion of HIV-infected in need), is it plausible that ART has reduced adult and/or infant mortality at a population level – from either vital registration or census or national household survey data?
**4. HIV/TB**
Can time trends in TB case notifications, estimated TB incidence, mortality, co-infection with HIV, and DOTS treatment outcomes be plausibly related to HIV prevalence, coverage of ART and combined TB/HIV interventions such as isoniazid (IPT) or cotrimoxazole prophylactic treatment (CPT)?
**5. PMTCT**
How has mortality among infants (less than 1 year including neonates) and children 1 to 4 years developed, at population level and in hospitals? To what extent can reductions plausibly be attributed to PMTCT uptake among women HIV-tested, given HIV infection rates in pregnant women?

### Data sources and triangulation methodology

Our analysis follows the standard approach for public health data triangulation, focusing on trends over time, by population (age/sex) group, in nationally aggregated data from health facilities assembled in the national Health Management Information System (HMIS), and from population-based health and demographic surveys and census. Each dataset is assessed for consistency, representativeness and quality over time, with subsequent triangulation among the different data sets focusing on the consistency of patterns over time and by population groups.

The main data sources were program records and patient registries from the HTC, ART, TB and PMTCT programs, two-yearly HIV sentinel surveillance among women attending ANC [31], annual data on morbidity and mortality registered in health facilities and hospitals assembled in the national HMIS, the 2007 Population Census [25] and 2007 Demographic and Health Survey (DHS) [26]. In addition, we used national-level estimates of incidence, prevalence, AIDS deaths and population size produced with the internationally agreed HIV epidemic model Spectrum, developed by UNAIDS and technical partners [32]. These databases are available at national and sub-national level, but since Swaziland is a small country and the socio-economic, demographic and health situation does not significantly differ across regions, this study focuses on data at national level. Data are disaggregated by age and sex where appropriate. There was no need for special ethical committee clearance because existing secondary data sources were used. The data sources, including their validity and completeness, are described in Table S1 in the online appendix.

An additional source considered was the vital statistics from the Department of Births, Marriages and Deaths. While potentially a uniquely detailed and continuous dataset on population-level mortality, however, concerns about varying completeness, representativeness and accuracy, as well as lack of collaboration with MoH led us to exclude this source from the triangulation.

### Analysis

Swaziland’s hospital monitoring information system captures in-patient admissions from all public hospitals, including the reason for admission (diagnosis based on ICD-9 coding system) and discharge status (e.g. dead, discharged). For the purpose of this study, in-patient case fatality rate (CFR) is defined as the proportion of patients admitted to hospital who died in a given year. Numbers of tuberculosis notifications, admissions and deaths were standardized according to estimated population size, and presented as rates per 100,000 population members per year. Infant population size was estimated according to the population age distribution as sampled by the 2007 DHS.

## Results

### Prevalence and Incidence

**Figure 1:**
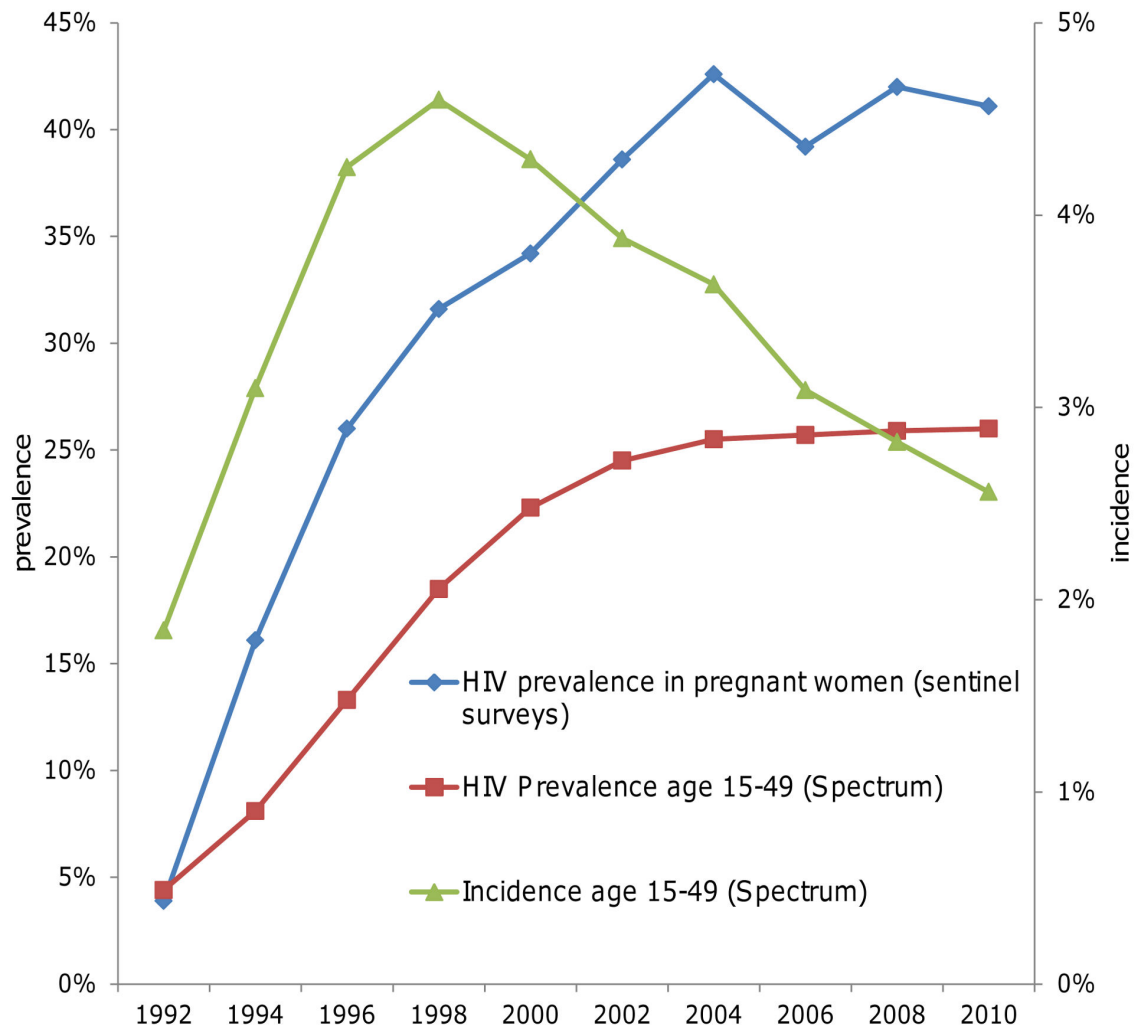
HIV prevalence and incidence over time.

Among pregnant women tested in HIV sentinel sero-surveillance, HIV prevalence increased from 3.9% in 1992 to a peak of 42.6% in 2004, after which prevalence has stabilized at just above 40% until 2010 (Figure 1) [31]. Using these data in the Spectrum model, SNAP with UNAIDS estimate that HIV incidence in adults aged 15-49 years had peaked at 4.6% in Swaziland in 1998, followed by a consistent downward trend to 2.6% in 2010, with adult prevalence in the overall adult population stable at around 26% since 2006 [32].

### HIV testing and counseling and coverage of antiretroviral therapy

**Figure 2:**
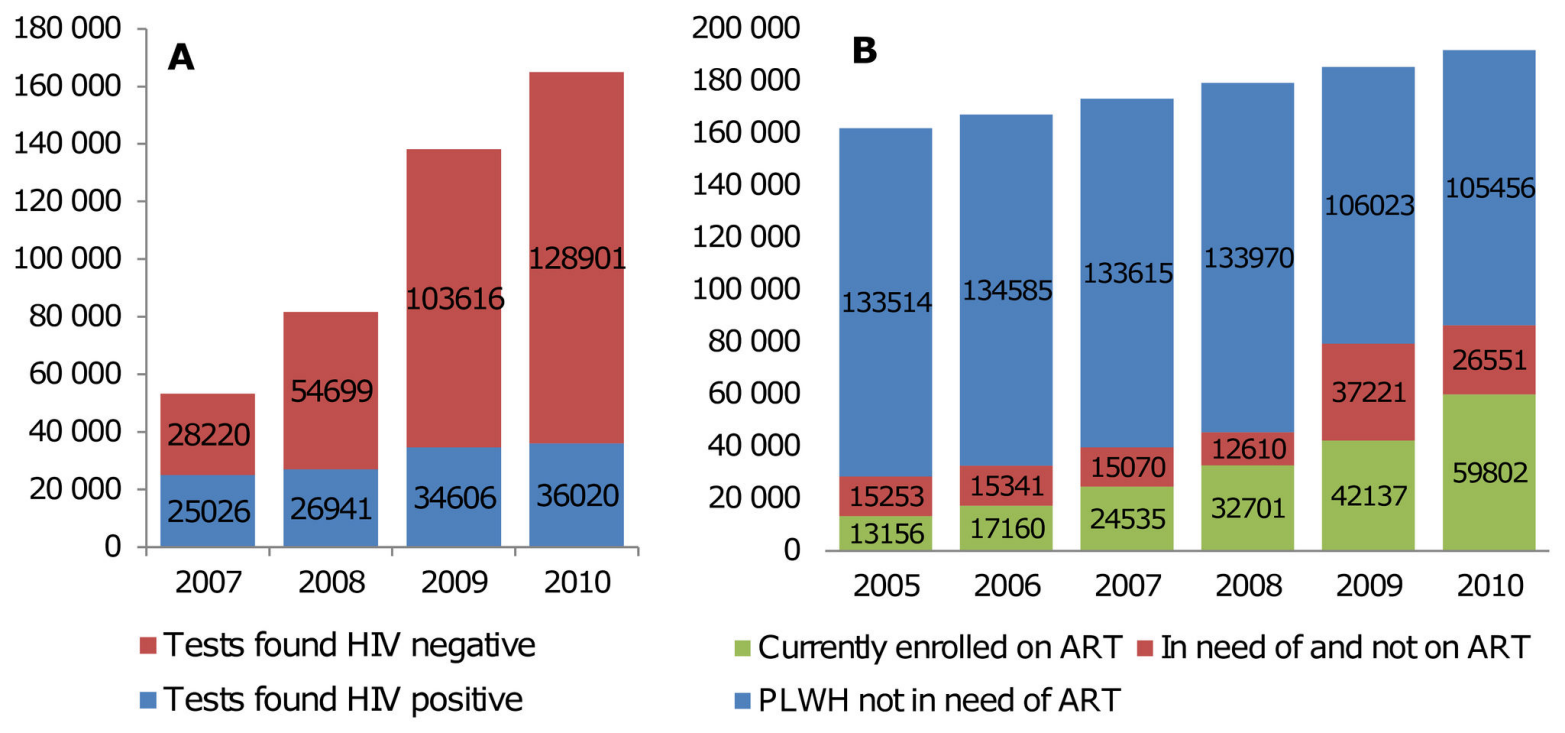
Uptake of HIV testing and counseling (A) and (B) estimated numbers of PLWH [32], according to ART eligibility/need of actual enrolment on ART, at the end of every year.

In 2010 alone, an estimated 244 per 100,000 adults 15 years and above received HIV testing and counseling in a HTC clinic, according to program records [33]. In 2006-7, 36% of women aged 15-49 and 17% of men aged 15-49 reported to have ever received an HIV test and to know its result [26]. The number of HIV tests administered increased from almost 60,000 in 2007 to more than 160,000 in 2010 (Figure 2A). The proportion of HIV tests with a positive (infected) result fell from 47% in 2007 to 22% in 2010. Swaziland has made substantial progress in providing ART to people in need, with estimated coverage increasing from 46% in 2005 to 72% [95% CI 67-76%] in 2010 [33], where coverage is defined as the ratio of people living with HIV/AIDS (PLWH) on treatment relative to the Spectrum-estimated number of PLWH who are eligible for treatment according to the national guidelines.

### Mortality estimates

Swaziland, like most sub-Saharan countries, does not have a well-functioning reliable system for registering births and deaths; therefore mortality trends can only be assessed from periodic household surveys, the ten-yearly national population census, and small-scale longitudinal research studies. Based on Census, the crude, all-age death rate increased from 7.6 to 18 per 1,000 from 1997 to 2007; or 2.8 to 16.8 per 1,000 for the 15-49 year age group. Over the same period, the estimated infant mortality rate increased from 78 to 107 per 1,000, while the under-5 mortality rate increased from 106 to 167 per 1,000 [25]. The 2007 DHS estimated age-specific adult (aged 15-49 years) mortality for the six years before the survey using sibling survivorship history. Mortality for men and women were estimated as 14.4 deaths per 1,000 person years [26] for the 2001-2007 period, consistent with the trend in Census data from 1997 to 2007. Furthermore, Spectrum estimated HIV/AIDS attributable mortality for adults aged 15-49 years at 12.8 per 1,000 person years in 2007 [32].

### In-patient case fatality rate

**Figure 3:**
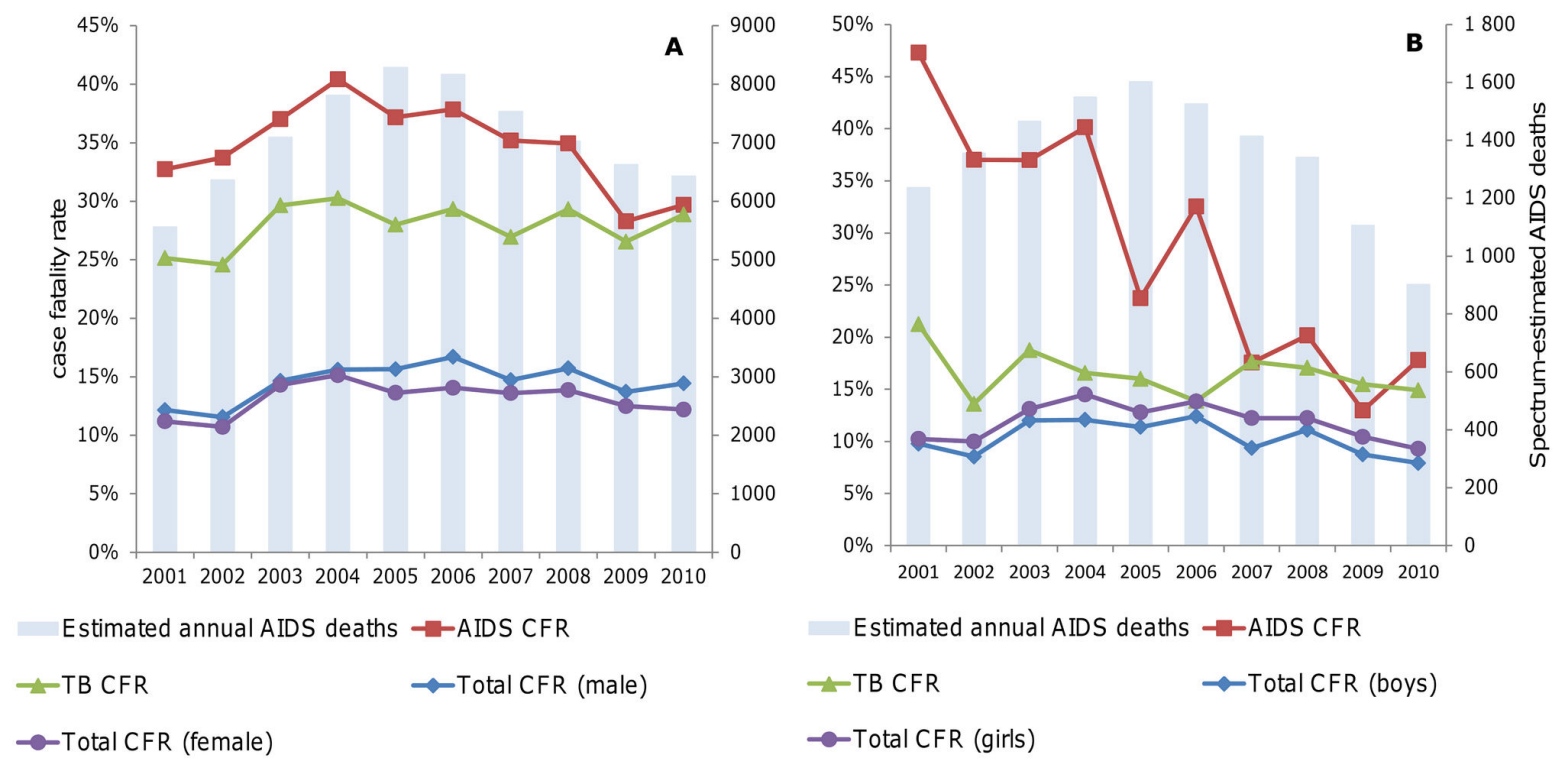
Case fatality rates among in-patients according to hospital cause-of-death coding registered in the health management information system, in relation to Spectrum-estimated AIDS deaths, for (A) adults (aged 15+) and (B) children (aged 0-14) [32].

In-patient case fatality rates for AIDS-attributed admissions as well as all-cause admissions in females aged 15 or older rapidly rose from 2001 to a peak in 2004-5, followed by a gradual decline until 2009 (Figure 3A). For children, case fatality rates for in-patients diagnosed with HIV/AIDS started declining from around 2004, with corresponding though less marked declines for inpatient deaths from all causes (Figure 3B). These declines coincided with, or slightly preceded, the start of roll-out of ART and PMTCT. For TB, the case fatality rate among adult in-patients remained at around 30% between 2004 and 2010, while it dropped slightly in children admitted with TB from 2007. All-cause case fatality rates were higher for adult males than females since 2005.

### All-cause admissions and deaths

**Figure 4:**
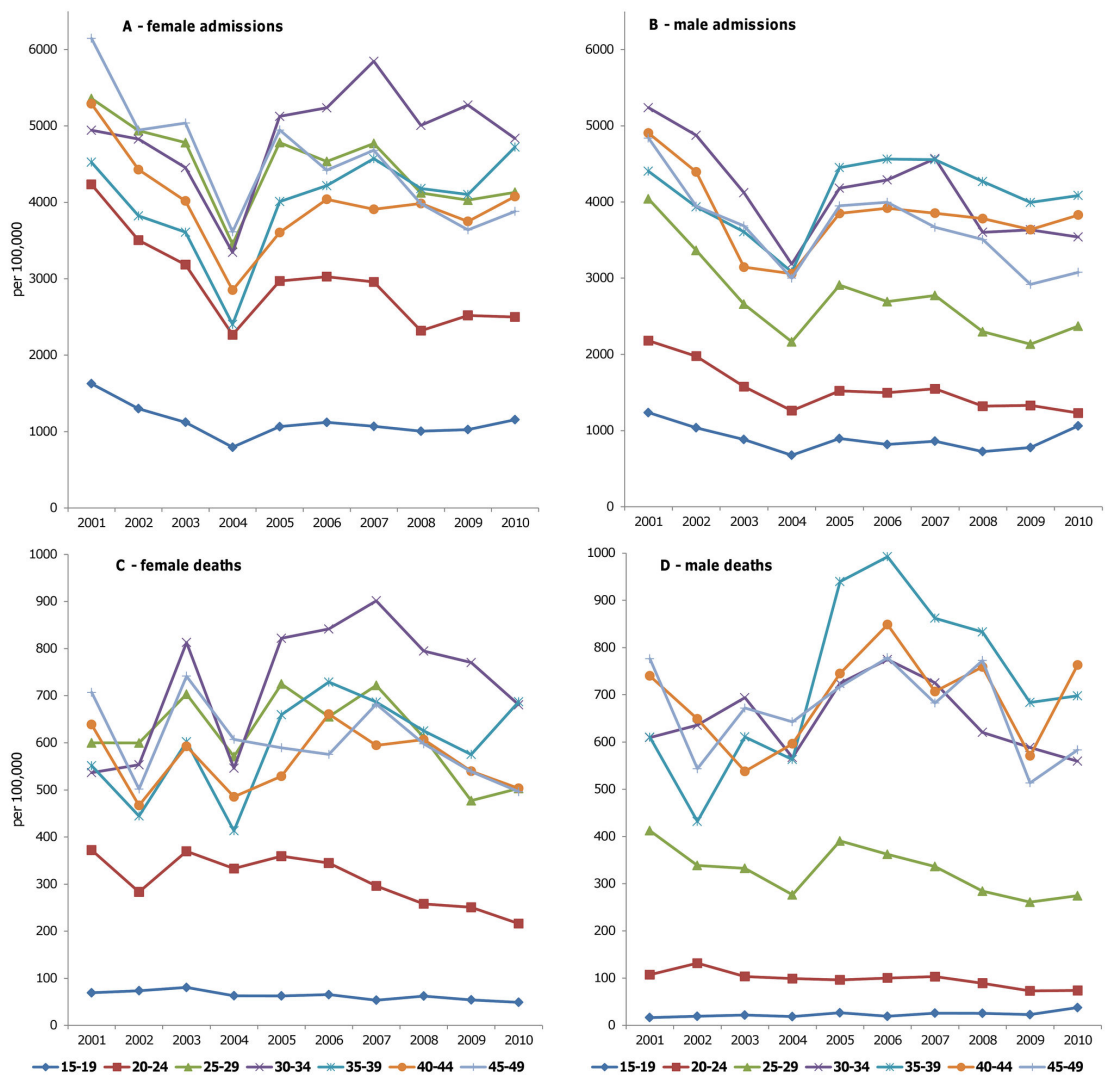
Population standardized number of hospital admissions for (A) females and (B) males aged 15-49 and population standardized number of hospital deaths for (C) females and (D) males aged 15-49.

Figure 4 shows population-standardized in-patient admissions and deaths, for males and females separately and for adults aged 15-49 by five-year age bands. In general, there are more admissions and deaths for females than for males, even though we excluded maternity-related admissions. Numbers of all-cause admissions have stabilized or slightly increased for most age groups since 2005, whereas numbers of deaths tended to decrease after the decentralization of ART in 2007-8 for women and men in all age groups.

### Retention and survival on antiretroviral therapy

**Figure 5:**
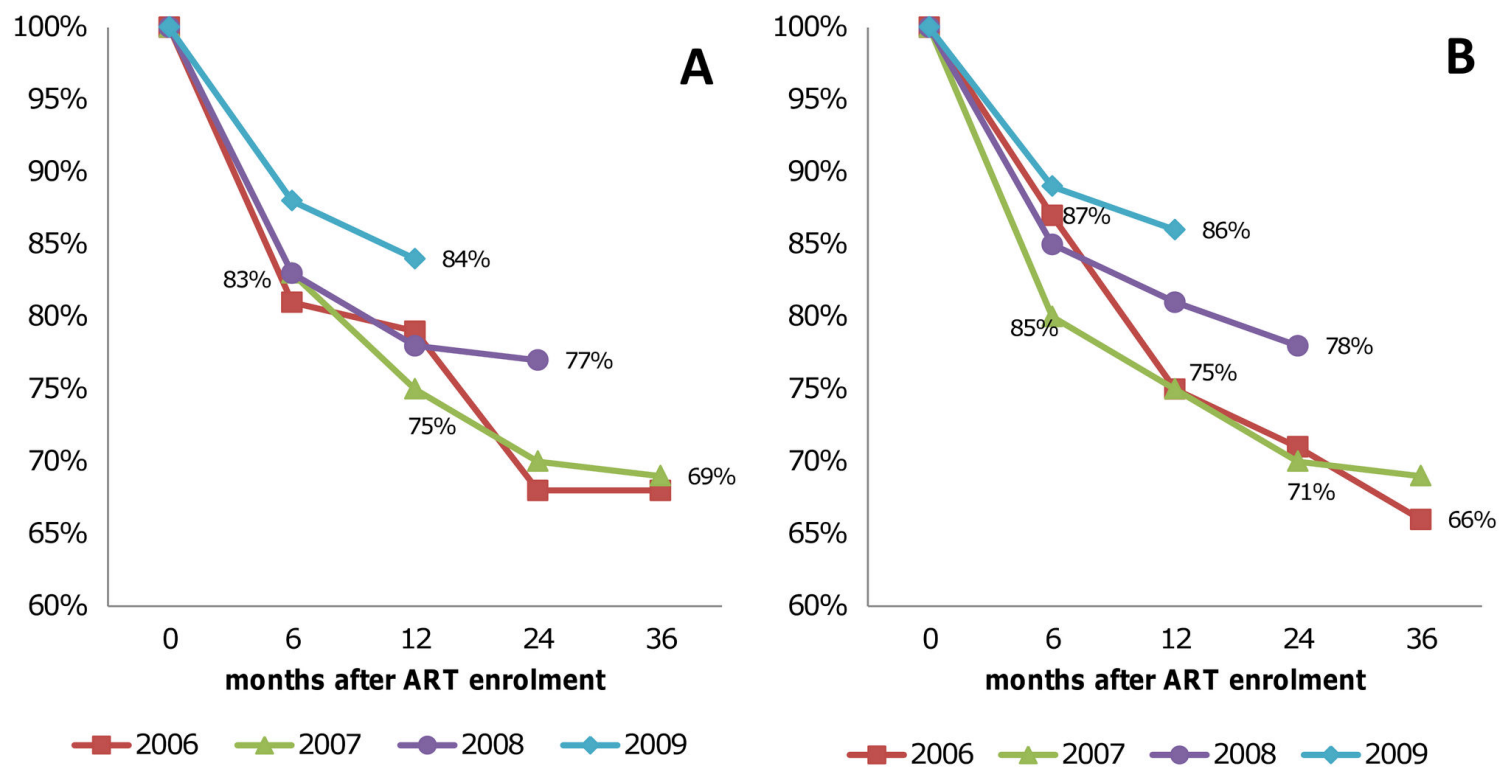
Proportion of patients retained over time after ART enrolment for (A) adults (aged 15+), and (B) children (0-14 years).

Based on the national ART patient registry, significant losses occur among both adult and pediatric patients enrolled on ART, especially within the first six months after enrolment on ART (Figure 5). These losses include deaths, loss-to-follow-up and patients stopping treatment. Patient retention rates improved over consecutive calendar years. For the cohort of adults enrolled in 2009, the 12-month retention was 84%, just below the WHO target of 85%, compared to only 75% for the cohort of adult patients enrolled in 2007. For children enrolled in 2009, the 12-month retention was 86%, above the target.

### Tuberculosis and TB/HIV co-morbidity

**Figure 6:**
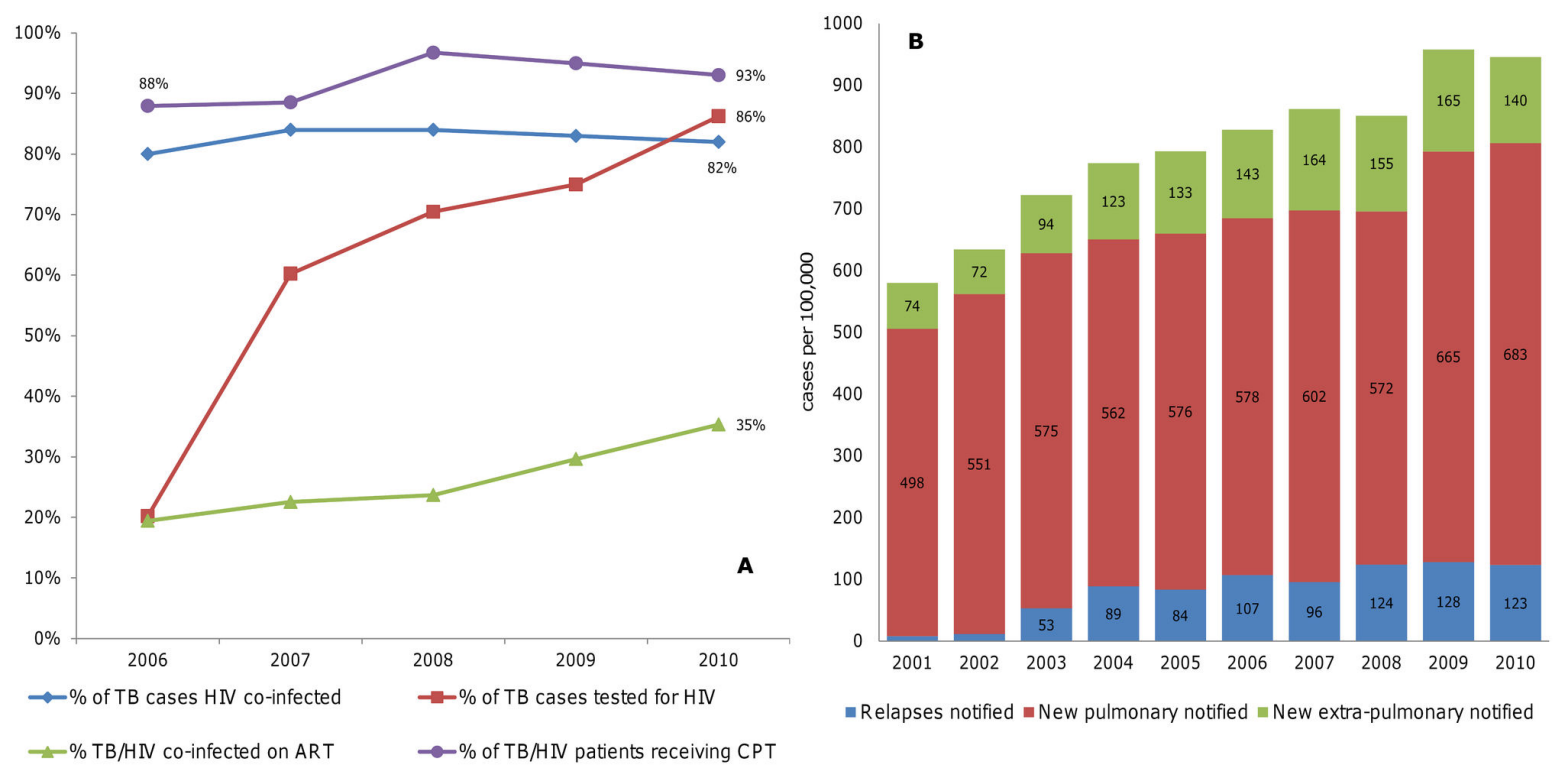
TB and HIV co-infection rates, coverage of TB/HIV interventions, according to NTCP registry (A) and TB notifications (B).

Swaziland remains among the countries with the highest TB disease incidence rates, estimated at 1,290 per 100,000 person years (95% CI 1,060-1,530) as of 2010 [34]. TB is a common and recurring opportunistic infection among HIV patients and ART can reduce the incidence and mortality of TB in HIV-infected people [35]. Around 80% of patients with active TB in Swaziland are also HIV-infected, and this proportion has been stable over the period 2006 to 2010 (Figure 6A). By 2010, according to TB program records, coverage of HIV screening among TB patients had reached 86%, coverage of CPT among TB/HIV co-infected patients 93%, and coverage of ART for TB/HIV co-infected patients 35%. Meanwhile, TB case notifications continued to increase until 2009, for both new pulmonary, new extra-pulmonary and relapse cases (Figure 6B). When disaggregated by age and sex, the largest and steepest increases in TB case notifications were in the age groups with highest HIV prevalence: men 35-44 years and women 25-34 years. The incidence of notified MDR-TB, which is associated with very high mortality even in the presence of ART, doubled from 9.8 new cases per 100,000 person years in 2007 to 20.1 new cases per 100,000 person years in 2010 [36].

### Prevention of mother to child transmission

**Figure 7:**
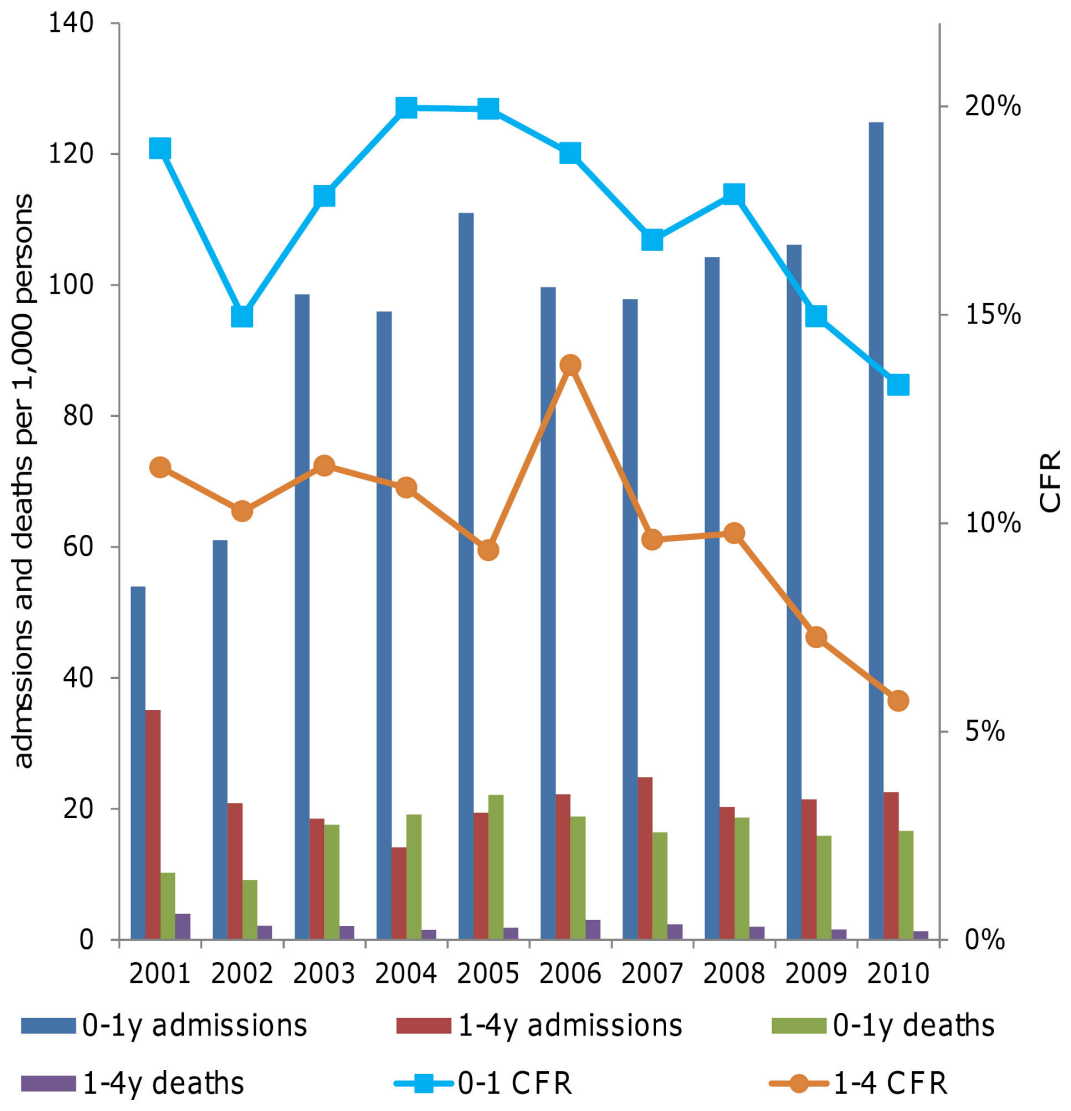
In-patient case fatality rate, population standardized hospital admissions and deaths, among infants (0-1 year) and children (1-4 years).

An estimated 11,000 infants were exposed to HIV in 2010, out of a total 35,000 births estimated for that year [32]. The percentage of HIV-exposed babies testing HIV-positive with DNA PCR methods dropped from 24% in 2007 to 12% in 2010. In parallel, in-patient all-cause case fatality rates fell sharply for both infants aged 0-1 year and children aged 1 to 4 years (Figure 7).

## Discussion

The lead and interest taken by the Ministry of Health of Swaziland facilitated this first comprehensive analysis of HIV and TB program outcomes and impact using triangulation with data available across systems and reporting levels.

The spread of HIV in Swaziland started later than in most other sub-Saharan countries, with a peak in HIV incidence in the late 1990s. Estimated adult incidence has declined since the early 2000s, before the roll-out of ART. A declining incidence and stable prevalence (since 2004) implies that morbidity and mortality has declined. It is however unclear to what extent these recent changes in incidence, mortality and morbidity can be attributed to ART, PMTCT and HTC services rolled out since 2003, to HIV prevention interventions, and/or to intrinsic epidemic dynamics.

### HIV testing and counseling and coverage of antiretroviral therapy

The halving of test positivity rates captured in the HIV testing and counseling database probably reflects the progressively increasing coverage of especially provider-initiated HIV testing and counseling among lower-risk populations.

### Impact of ART on mortality and morbidity

Census all-cause mortality estimates show a drastic increase between 1997 (just before the peak in HIV incidence) and 2007 (close to the estimated peak in mortality due to AIDS). Without data for intermediate and subsequent years, however, as yet not much can be inferred about the effect of ART on mortality.

As a proxy for population-level mortality trends, we investigated in-patient case fatality rates. Early evidence of the impact of the recent health sector responses is suggested by on-going declines in in-patient case fatality rates among infants (Figure 7) and children (Figures 3 and 7). Trends for adults are also somewhat suggestive of program impact (Figure 3). These trends are similar to those observed in HIV and TB impact assessments in Botswana [15].

The higher case fatality rate for adult males compared to adult females from 2005 could be indicative of differential access to treatment, with females accessing health facilities more frequently and showing higher ART coverage than men (79% vs. 58% of PLWH in need in 2010). The steep decrease in number of hospital admissions from 2001 to 2004 may partly reflect that, before ART became available, very sick AIDS patients occupied beds for longer, resulting in lower availability of beds for new patients. The high in-patient case fatality rate of AIDS patients could also have served as a psychological deterrent for patients to go to hospital.

As the Swaziland ART program improved and expanded over time, consecutive yearly cohorts of patients enrolled into ART showed improved retention during the first twelve months of treatment, but long-term retention is still poor. The persistent loss during the first six months of treatment is likely explained by late presentation for HIV/AIDS care, with up to 56% of patients having progressed to WHO clinical stage 3 or 4 by time of ART enrolment in 2010 [37]. Interventions that may contribute to progressively improving retention of patients include better adherence counseling services, simplified ART regimens with less pill burden and/or less severe side effects, and improved patient tracking and tracing systems.

A 2004 study used an undertaker’s data on numbers of funerals and coffin sales to infer changing patterns of mortality in Swaziland from late 1998 to early 2002 [38]. Significant increases in numbers of registered funerals were found in the age group 0-4 years, from around 300 in 1999 to 900 in 2001, and for the age group 20-49 years, from around 400 in 1999 to 900 in 2001. This proved a good source to assess mortality trends, since the specific undertaker operated nationally and owned a large market share. Also in rural Malawi, coffin sales and funerals registered by undertakers proved a useful indicator for time patterns in AIDS-related mortality [18]. Given Swaziland’s lack of functioning Vital Registration, trends in funeral and coffin sales could similarly be a useful data source to add to future triangulations, especially when including a before-ART baseline period.

Two large initiatives recently launched in Swaziland, the Swaziland HIV Incidence Measurement Survey (SHIMS) and the Maximizing ART for Better Health and Zero New Infections (MaxART) [39,40] project, should help improve estimates of HIV and TB incidence, their trends and program impact between now and 2014. First findings from SHIMS estimated national HIV incidence at 2.4% per annum and national HIV prevalence at 31% for the 18-49 age group, for 2012 [41]. Population-level data on all-cause mortality from the forthcoming 2017 population census and 2013/14 DHS will provide more conclusive evidence of the impact of ART on mortality trends.

### Tuberculosis and HIV co-infection

In contrast to recent declines in TB/HIV co-infection rates in Botswana, from 80% in 2003 to 66% in 2009 [15], and Malawi, from 70% in 2006 to 60% in 2009-10 [42], the TB/HIV co-infection rate has stayed level at around 80% in Swaziland from 2006 to 2010. Since these three countries scaled-up ART, PMTCT and HTC over roughly the same period, the difference may rather be explained by the difference in the underlying HIV epidemics, with HIV prevalence having stabilized and started to decline several years earlier in Malawi and Botswana than in Swaziland. At the other extreme, neighboring South Africa documented an ongoing increase in TB/HIV co-infection rates, from 52% in 2005, to 60% in 2010, and 65% in 2011 [34], which may be explained by a comparatively lower ART coverage, at only 36% [95% CI 30-43%] of people living with HIV/AIDS in need in 2008 [43].

TB notifications by the national TB program have historically under-reported real numbers of TB cases. According to WHO estimates, the increase in notifications from 2001 to 2009 parallels an increase in total TB disease incidence, while the case detection rate has been stable at around 70% [34]. The full impact of increased priority given recently to TB control, with strengthened case finding, improved diagnostic methods and reporting systems; routine screening for TB of all HIV patients and all patients visiting out-patient departments since 2009; initiation of all HIV-infected patients on INH prophylactic therapy to prevent TB since 2011 [44,45]; and recent expanded ART enrolment for all TB/HIV co-infected patients, as per Swaziland’s 2009 revised ART policy, should become apparent in NTCP data over coming years.

### Prevention of mother to child transmission

Assessing the impact of the overall PMTCT program on the mortality of infants and children aged one to four is challenging, but the observed downward all-cause hospital case fatality trends for these groups are encouraging. Infant in-patient admissions increased since 2007 – this is likely a result of the Integrated Management of Acute Malnutrition program that started in 2007, where the focus for treating malnourished children was shifted to hospitals [46]. An evaluation of the effectiveness of the national PMTCT program, led by the Ministry of Health (MoH) that focuses on infant outcomes at 6-8 weeks post-partum, started in 2012 and is on-going [9,47].

The steep decline in AIDS case fatality rates among admitted children (from 40% in 2004 to 17% in 2010) may reflect an impact of ART among infants and children – or alternatively, be an effect of expanding coverage of HIV diagnosis, including among children previously not recognized as having HIV/AIDS.

### Recommendations

The current triangulation has enabled the national programs to understand that showing impact is possible. Bringing together all in-country partners to work on this assessment proved to be a main challenge. Broader processes may be needed to facilitate effective partnerships, co-investment in common M & E frameworks, and country-led platforms of strategic information that international donors also support and use [48,49].

National authorities adopted the following recommendations based on the final study report [22]:

1 To further strengthen collaboration between TB and HIV programs, including a progressive linkage and integration of monitoring and reporting systems.
2 To review and strengthen the health management and information system (HMIS) and underlying data collection systems, for improved data quality. This process is on-going, notably with an upgrading of the current cause of disease and death classification guidance, from ICD-9 to ICD-10.
3 To establish mechanisms of collaboration between the departments of Births, Marriages and Deaths (BMD), and M & E in the Health Sector: This should ensure information sharing and accurate vital registration of deaths − with causes classified according to the conditions diagnosed in health facilities, hospitals or by the ART and TB programs. The committee currently reviewing the HMIS indeed includes members from the BMD to ensure collaboration.
4 For the ART program to further strengthen tracking, recording and tracing of patients enrolled on ART, to improve understanding and actual retention of patients.

### Limitations

The limitations of this study are two-fold: the first being related to the triangulation approach and second, the sources of data used.

Limitations inherent to public health triangulation [10] include that the data used were not originally collected for a systematic impact evaluation, notably they lack comparison/control groups. As a result, associations observed between intervention scale-up and health response (over time, by population group or among regions) do not necessarily indicate causal relationships.

For data sources used, the key limitations are summarized in Table S1 in the online appendix. Like other low-income African countries, Swaziland’s national health monitoring and information system (HMIS) suffers gaps and inconsistencies in data (due to changing, sub-optimal and sometimes parallel data collection, entry and reporting systems and tools), delays in reporting from health facility to regional and national levels, and aggregation of data at the national level which results in the loss of detailed data needed for in-depth analysis. A major shortcoming of the HMIS is the lack of unique patient identifiers, making linkage across programs impossible. These weaknesses probably reduced the power to assess ART and PMTCT impact from hospital in-patient data. In addition to these general problems with the HMIS, there are challenges with clinical diagnosis, in particular cause-of-death recording. For the national ART program, a 2009 external data quality assessment reported uncertainty about the precise number of people on ART, with some over-reporting identified in several sites [50].

For 2010, the NTCP reported that only 35% of HIV co-infected TB patients newly diagnosed with TB were receiving ART – much below the overall 70% coverage of ART among all people living with HIV. This inconsistency illustrates that data from SNAP and NTCP are not synchronized properly. Efforts to harmonize these two parallel systems are being discussed.

## Conclusion

Seven years after the launch of its ART, PMTCT and HTC programs, Swaziland is demonstrating effective intervention coverage and early evidence of impact on AIDS-related mortality in hospitals. This assessment could not yet capture the full impact of the 2009 revision of HIV control policies to scale-up provider-initiated testing for HIV, screening HIV patients for TB, screening TB patients for HIV, and ART for a broadened set of patients including all TB/HIV co-infected patients. Future similar triangulations, based on more years of progressively better-quality and population-based data, are expected to show more prominently and more conclusively the significant impact that the national HIV program, with support from partners, has started to realize.

## Supporting Information

Table S1 Data Sources and Limitations.
(DOCX)
