# Supplementary material for: Outcomes and Impact of HIV Prevention, ART and TB Programs in Swaziland – Early Evidence from Public Health Triangulation
Source: PLoS One. 2013 Jul 26;8(7):e69437. doi: 10.1371/journal.pone.0069437 (PMC3724860; doi:10.1371/journal.pone.0069437)
Supplement: Table S1 — Data Sources and Limitations. (DOCX) [file pone.0069437.s001.docx]

**Table S1.** Data Sources and Limitations

| **Data source** | **Variables** | **Limitations** |
| --- | --- | --- |
| 1. ART program information system | - People newly enrolled on ART - People currently on ART - People ever enrolled on ART - Cohort outcomes: alive and on ART, died, stopped, lost to follow up, restarted | - Frequent updates/revisions of electronic and paper systems compromise data quality. [[1](#_ENREF_1)] - High loss-to-follow-up raises concern about potential double-entries of the same patient at multiple sites. [[2](#_ENREF_2)] - Up to 2008 data on all children were aggregated; split into <1, 1-4 and 5-14 years since 2009. |
| 1. PMTCT program information system | - Pregnant women tested and counseled for HIV - HIV-infected women receiving ARV prophylaxis to reduce MTCT - HIV-exposed infants (<1 year) tested for HIV infection diagnosed with DNA PCR - HIV-exposed infants found HIV-infected through DNA PCR | - Aggregated monthly – no patient-level data available. - Number of infants enrolled on ART is only recorded since 2009. |
| 1. HTC program information system | - HIV tests administered - HIV test results | - Aggregate numbers considered reliable since 2007 only; national electronic database since 2009. - Only number of tests known, not number of people tested. Poorly defined variable “Retest” used to derive number of people tested. |
| 1. TB program | - TB notifications, separately for new sputum smear-positive, new sputum smear-negative, smear not done, extra-pulmonary TB and relapse cases. - TB patients tested for HIV and number found HIV co-infected, separately for each category of notifications. - Known HIV co-infected TB patients receiving ART and CTX. - DOTS outcome data (cured, completed, died, defaulted, stopped, transfer out), separately by TB form and HIV status. - MDR-TB cases | - Reliable program data is only available from 2006. - Until 2013, no electronic national information system. |
| 1. HMIS hospital registry | Hospital admissions, with   - Diagnosis (ICD-9 coding system) - Discharge status (e.g. death, referred to other facility or sent home) | - Delays in reporting from health facility to regional and national level. - Data recorded as cases instead of unique patients. |
| 1. HIV Sentinel surveillance | - HIV prevalence in pregnant women attending Antenatal Clinics | - Pregnant women are not fully representative of the general population, with direction and magnitude of biases changing over the course of the HIV epidemic [[3](#_ENREF_3)]. - No electronic database for continuous reference and use. |
| 1. Swaziland Population Census of 1997 and 2007 | - Estimated mortality numbers and rates | - Once every ten years, next census planned for 2017. |
| 1. Demographic and Health Survey (DHS) 2006-7 | - HIV prevalence in adults 15-49 years - Proportion of adults reporting to have received a HIV test (ever and in last year) and to know its result - HIV related knowledge, attitudes and behavior. | - Only one DHS so far, next DHS planned 2013. |
| 1. Spectrum HIV epidemic modeling | - Population size and growth - HIV Prevalence, Incidence and mortality, as rates and numbers - People living with HIV/AIDS in need of ART and ART coverage as % of need - HIV-infected pregnant women in need of PMTCT | - Incidence modeling is not yet a robust tool – age distribution in HIV population assumed constant over the years. - Model outputs depend on the quality of the input data. - No module for TB incidence and TB/HIV epidemiology. |

1. World Health Organization, United States Government/President’s Emergency Plan for AIDS Relief, Swaziland Ministry of Health and Social Services, Swaziland National AIDS Program. (2010) Report of the Swaziland ART Program Review.

2. Swaziland Ministry of Health and Social Services, World Health Organization (2010) Assessment of loss-to-follow-up and associated factors among ART clients in Swaziland. Swaziland Ministry of Health website. Available at:

<http://www.gov.sz/images/stories/Health/ltfu_complete%20dec%2021.pdf> (Accessed 21 June 2013).

3. Gouws E, Mishra V, Fowler TB (2008) Comparison of adult HIV prevalence from national population-based surveys and antenatal clinic surveillance in countries with generalised epidemics: implications for calibrating surveillance data. Sexually Transmitted Infections 84: i17-i23.
